# Combination of platelet count and lymphocyte to monocyte ratio is a prognostic factor in patients undergoing surgery for non-small cell lung cancer

**DOI:** 10.18632/oncotarget.18336

**Published:** 2017-06-01

**Authors:** Wei Liu, Minwen Ha, Nanchang Yin

**Affiliations:** ^1^ Department of Medical Oncology, The First Affiliated Hospital of Jinzhou Medical University, Jinzhou, China; ^2^ Department of Thoracic Surgery, The First Affiliated Hospital of Jinzhou Medical University, Jinzhou, China

**Keywords:** platelet count, lymphocyte to monocyte ratio, non-small cell lung cancer, prognosis

## Abstract

The aim of this study was to investigate the usefulness of a novel inflammation-based prognostic system, called COP-LMR (combination of platelet count and lymphocyte to monocyte ratio), for predicting postoperative survival of patients with non-small cell lung cancer (NSCLC). COP-LMR was calculated on the basis of the obtained data. Patients with both an elevated platelet count (PLT) (>30 × 104mm-3) and a low LMR (<3.6) were assigned a score of 2, and patients with one or none of the parameters were assigned a score of 1 or 0, respectively. A total of 1120 patients who underwent complete resection were enrolled in this study. Multivariate analysis revealed that COP-LMR is an independent prognostic factor for disease-free survival (DFS) (*P*<0.001) and overall survival (OS) (*P*<0.001). Kaplan-Meier analysis and the log-rank test revealed that COP-LMR stratified the patients into 3 independent groups (*P*<0.001). In conclusion, COP-LMR is a potential prognostic biomarker in patients undergoing surgery for NSCLC.

## INTRODUCTION

Lung cancer is the leading cause of cancer-related mortality worldwide [1]. Radical surgery is the primary treatment strategy for lung cancer. Despite advances in lung cancer surgery, the 5-year survival rate of patients with lung cancer is still unsatisfactory. The median survival period for lung cancer in China is only 22.7 months [2]. Until now, there has been no ideal method to predict the survival of lung cancer patients. Therefore, it is urgent and essential to identify a reliable prognostic factor for lung cancer patients who underwent surgery. It would enable good risk stratification by clinicians and individualized management of lung cancer patients.

Recent studies have revealed that systemic inflammatory responses play an important role in cancer progression [3, 4]. Some of the identified inflammation-based prognostic markers include the Glasgow Prognostic Score (GPS) [5], platelet to lymphocyte ratio (PLR) [6], neutrophil to lymphocyte ratio (NLR) [7], lymphocyte to monocyte ratio (LMR) [8] and thrombocytosis [9]. Among these useful and convenient systems, the GPS is regarded as a prognostic milestone from which all of the other systems were derived [5]. The GPS is based on the estimation of concentrations of two acute proteins: C-reactive protein and albumin; these proteins reflect the magnitude of inflammatory response arising from tumor-host interactions [10]. Combinations of other types of inflammation-based markers are prognostically useful and serve as adjuncts to the GPS. Therefore, the estimation of reactive thrombocytosis and LMR might also be useful for predicting prognosis.

In this study, we analyzed the prognostic utility of a novel inflammation-based prognostic system, COP-LMR, in patients undergoing surgery for NSCLC.

## RESULTS

### Characteristics of patients

Table 1 shows the distribution of clinicopathological parameters of the studied patients divided into 3 groups according to COP-LMR. A total of 1120 patients were enrolled in our study. A total of 728 patients (65.0%) were men, and 392 patients (35.0%) were women. The median age of the participants was 60 years, and the age range was 22 years to 85 years. According to the seventh edition of the tumor-node-metastasis (TNM) classification, 477 patients were stage I, 237 patients were stage II, and 406 patients were stage IIIA. We divided the patients into 3 different groups on the basis of the cut-off values: 523 patients with COP-LMR = 0, 459 patients with COP-LMR = 1, and 138 patients with COP-LMR = 2.

**Table 1:** Association of COP-LMR with the clinicopathological characteristics of patients with NSCLC

Variables	COP-LMR = 0 *n*(%)	COP-LMR = 1 *n*(%)	COP-LMR = 2 *n*(%)	*P* value
**Age (year)**				0.885
**≤60**	283 (54.1)	243 (52.9)	76 (55.1)	
**>60**	240 (45.9)	216 (47.1)	62 (44.9)	
**Sex**				<0.001
**Female**	226 (43.2)	126 (27.5)	40 (30.0)	
**Male**	297 (56.8)	333 (72.5)	98 (70.0)	
**Smoking status**				<0.001
**Yes**	304 (58.1)	335 (73.0)	96 (69.6)	
**No**	219 (41.9)	124 (27.0)	42 (30.4)	
**Tumor location**				0.093
**Left**	224 (42.8)	166 (36.2)	52 (37.7)	
**Right**	299 (57.2)	293 (63.8)	86 (62.3)	
**Lesion type**				<0.001
**Peripheral**	413 (79.0)	295 (64.3)	94 (68.1)	
**Central**	110 (21.0)	164 (35.7)	44 (31.9)	
**Resection type**				<0.001
**Pneumonectomy**	44 (8.4)	62 (13.5)	28 (20.3)	
**Lobectomy**	479 (91.6)	397 (86.5)	110 (79.7)	
**Histological subtype**				<0.001
**SqCC**	198 (37.9)	245 (53.4)	82 (59.4)	
**Adenocarcinoma**	261 (49.9)	156 (34.0)	34 (24.6)	
**Others**	64 (12.2)	58 (12.6)	22 (16.0)	
**Lymph node metastasis**				0.032
**Yes**	222 (42.4)	229 (49.9)	56 (40.6)	
**No**	301 (57.6)	230 (50.1)	82 (59.4)	
**Pathological stage**				<0.001
**I**	275 (52.6)	164 (35.7)	38 (27.6)	
**II**	74 (14.1)	109 (23.8)	54 (39.1)	
**IIIA**	174 (33.3)	186 (40.5)	46 (33.3)	

Abbreviations: COP-LMR, combination of preoperative platelet count and lymphocyte to monocyte ratio; NSCLC, non-small cell lung cancer; SqCC, Squamous cell carcinoma. *P* value <0.05 is statistically significant.

### Receiver operating characteristic curve for prediction of overall survival

The optimum cut-off values for the preoperative platelet count (PLT) and LMR for survival prediction were determined from the receiver operating characteristic curve (ROC) curves. For PLT, the optimum cut-off point was 29.5 ×104 mm-3 with a maximum joint sensitivity of 45.4%, specificity of 73.8%, and an area under the ROC curve (AUC) of 0.576 (95% CI: 0.543-0.610). Therefore, the recommended cut-off value of preoperative PLT was 30.0 × 104 mm-3. For LMR, the optimum cut-off point was 3.6 with a maximum joint sensitivity of 52.7% and specificity of 71.1% on the ROC plot. The AUC was 0.641 (95% CI: 0.609-0.673) (Supplementary Figure S1). For each biomarker, the patients were classified into two groups (PLT: < 30.0 ×104 mm-3 and ≥ 30.0 ×104 mm-3; LMR: < 3.6 and ≥ 3.6).

**Figure 1:**
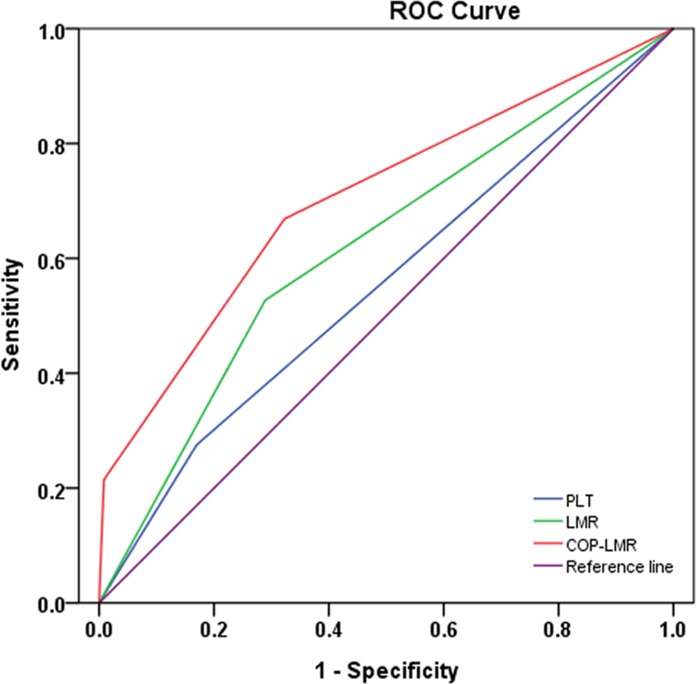
ROC curves used to evaluate the predictive accuracy of 5-year survival rates The AUC for PLT, LMR and COP-LMR was 0.553, 0.619 and 0.705.

Next, the AUC for 5-year OS was measured and compared with the method established by DeLong et al [11]. The AUC for PLT, LMR and COP-LMR was 0.553 (95% CI: 0.519-0.586), 0.619 (95% CI: 0.586-0.652) and 0.705 (95% CI: 0.675-0.735), respectively (Figure 1), indicating that COP-LMR was superior to PLT or LMR as a predictive factor in NSCLC patients undergoing surgery.

### Relationship between COP-LMR and clinicopathological and clinicolaboratory variables

The relationships between COP-LMR and clinicopathological and clinicolaboratory variables are shown in Tables 1 and 2. Our results revealed that COP-LMR was associated with sex (*P* < 0.001), smoking status (*P* < 0.001), lesion type (*P* < 0.001), resection type (*P* < 0.001), histological subtype (*P* < 0.001), lymph node metastasis (*P* = 0.032), and pathological stage (*P* < 0.001). Furthermore, COP-LMR was also associated with maximum tumor diameter (*P* < 0.001), WBC count (*P* < 0.001), hemoglobin (Hb) (*P* < 0.001), albumin (*P* < 0.001), alkaline phosphatase (ALP) (*P* = 0.005), D-dimer (*P* = 0.008), fibrinogen (*P* < 0.001) and survival period (*P* < 0.001). In contrast, COP-LMR displayed no association with age, tumor location or lactate dehydrogenase (LDH) level.

**Table 2:** Association of COP-LMR with the clinicolaboratory characteristics of patients with NSCLC

Variables	COP-LMR=0(*n*=523)	COP-LMR=1(*n*=459)	COP-LMR=2(*n*=138)	*P* value
**Age (year)**	60.7±9.4	59.9±9.5	59.8±8.8	0.351
**Maximum tumor diameter (cm)**	3.7±1.6	4.6±2.1	6.0±2.8	<0.001
**WBC count (×10**3**/μL)**	6.4±1.7	7.2±1.7	7.5±1.6	<0.001
**Hb (gL^−1^)**	141.4±14.7	139.0±16.5	130.3±16.2	<0.001
**Albumin (gL^−1^)**	43.8±3.9	42.4±4.2	40.7±4.3	<0.001
**LDH (UL^−1^)**	181.5±41.0	182.1±55.1	180.4±42.1	0.924
**ALP (UL^−1^)**	72.7±21.2	77.7±28.5	78.5±34.6	0.005
**D-dimer (mgL^−1^)**	0.19±0.23	0.19±0.14	0.25±0.36	0.008
**Fibrinogen (gL^−1^)**	3.3±0.8	3.9±0.9	4.5±1.0	<0.001
**Survival period (m)**	49.0	42.0	26.5	<0.001

Abbreviations: COP-LMR, combination of preoperative platelet count and lymphocyte to monocyte ratio; NSCLC, non-small cell lung cancer; WBC, white blood cell; Hb, hemoglobin; LDH, lactate dehydrogenase; ALP, alkaline phosphatase; m, month. Survival period, median survival. *P* value <0.05 is statistically significant.

### Relationship between COP-LMR and survival

The median follow-up time for the 1120 patients was 45.0 (range: 2-96) months. A total of 643 patients died during the observation period. The 5-year OS rate was 44.5%. By univariate analysis, we found that COP-LMR was correlated with DFS (*P* < 0.001) and OS (*P* < 0.001) (Table 3). Factors including lesion type, resection type, pathological stage, LDH, ALP, Hb, albumin, WBC count, PLT, D-dimer, fibrinogen and LMR were also significant for DFS and OS. The 13 clinical characteristics that were found significant (*P* < 0.05) by univariate analysis were assessed by a multivariate analysis. Our results revealed that preoperative COP-LMR was associated with DFS (*P* < 0.001) and OS (*P* < 0.001), while PLT and LMR were not significant factors in predicting DFS and OS. Furthermore, pathological stage, Hb and D-dimer were also revealed as independent prognostic factors (Table 4). The multivariate analysis also showed that COP-LMR served as an independent prognostic factor in squamous cell carcinoma (SqCC) or adenocarcinoma (Supplementary Tables S1–S4).

**Table 3:** Univariate analysis for DFS and OS for all NSCLC patients

Variables		*P* value	DFS HR (95 % CI)	*P* value	OS HR (95 % CI)
**Age (≤60/>60)**		0.633	0.963(0.825-1.125)	0.458	0.943(0.808-1.101)
**Sex (female/male)**		0.703	0.969(0.822-1.141)	0.286	0.915(0.777-1.077)
**Smoking status (yes/no)**		0.432	1.068(0.905-1.259)	0.110	1.144(0.970-1.350)
**Tumor location (left/right)**		0.909	1.009(0.862-1.182)	0.947	1.005(0.858-1.178)
**Lesion type (central/peripheral)**		0.006	1.264(1.070-1.492)	0.004	1.276(1.081-1.507)
**Resection type (pneumonectomy/lobectomy)**		0.034	1.275(1.019-1.597)	0.035	1.274(1.017-1.595)
**Histological subtype (SqCC/non-SqCC)**		0.870	1.013(0.868-1.183)	0.894	0.990(0.848-1.155)
**Pathological stage (IIIA/I, II)**		<0.001	2.507(2.145-2.930)	<0.001	2.484(2.126-2.903)
**LDH (≥174.0/<174.0 UL^−1^)**		0.036	1.180(1.015-1.377)	0.049	1.168(1.001-1.364)
**ALP (≥71.0/<71.0 UL^−1^)**		0.008	1.233(1.056-1.439)	0.005	1.246(1.067-1.455)
**Hb (≥130.5/<130.5 gL^−1^)**		<0.001	0.662(0.560-0.783)	<0.001	0.677(0.572-0.801)
**Albumin (≥44.9/<44.9 gL^−1^)**		<0.001	0.682(0.574-0.810)	<0.001	0.691(0.582-0.822)
**WBC count (≥7.8/<7.8× 10**3 **mm**-3)		<0.001	1.343(1.139-1.583)	0.001	1.328(1.127-1.565)
**PLT (≥300/<300 ×109L^−1^)**		<0.001	1.435(1.206-1.706)	<0.001	1.437(1.208-1.706)
**D-dimer (≥0.1/<0.1 mgL^−1^)**		<0.001	1.442(1.235-1.684)	<0.001	1.359(1.163-1.588)
**Fibrinogen (≥3.6/<3.6 gL^−1^)**		<0.001	1.405(1.203-1.641)	<0.001	1.451(1.242-1.695)
**LMR (≥3.6/<3.6)**		<0.001	0.537(0.460-0.627)	<0.001	0.532(0.456-0.621)
**COP-LMR (1, 2/0)**		<0.001	1.915(1.646-2.230)	<0.001	1.909(1.641-2.223)

Abbreviations: DFS, disease-free survival; OS, overall survival; HR, hazard ratio; CI, confidence interval; NSCLC, non-small cell lung cancer, SqCC, squamous cell carcinoma; LDH, lactate dehydrogenase; ALP, alkaline phosphatase; Hb, hemoglobin; WBC, white blood cell; PLT, platelet count; LMR, lymphocyte to monocyte ratio; COP-LMR, combination of preoperative platelet count and lymphocyte to monocyte ratio. HR was calculated with reference to the last category. *P* value <0.05 is statistically significant.

**Table 4:** Multivariate analysis for DFS and OS for all NSCLC patients

Variables		*P* value	DFS HR (95 % CI)	*P* value	OS HR (95 % CI)
**Lesion (central/peripheral)**		0.255	1.116(0.924-1.347)	0.319	1.102(0.911-1.333)
**Resection type (pneumonectomy/lobectomy)**		0.703	1.050(0.818-1.348)	0.870	1.021(0.793-1.314)
**Pathological stage (IIIA/I, II)**		<0.001	2.395(2.045-2.807)	<0.001	2.416(2.063-2.829)
**LDH (≥174.0/ <174.0 UL^−1^)**		0.056	1.199(0.995-1.407)	0.066	1.161(0.990-1.361)
**ALP (≥71.0/<71.0 UL^−1^)**		0.378	1.077(0.914-1.268)	0.238	1.104(0.937-1.301)
**Hb (≥130.5/<130.5 gL^−1^)**		0.001	0.745(0.623-0.890)	0.001	0.743(0.621-0.889)
**Albumin (≥44.9/<44.9 gL^−1^)**		0.065	0.828(0.698-1.014)	0.074	0.844(0.701-1.016)
**WBC count (≥7.8/<7.8× 10**3 **mm**-3)		0.069	1.199(0.995-1.426)	0.110	1.152(0.968-1.372)
**D-dimer (≥0.1/<0.1 mgL^−1^)**		0.014	1.223(1.041-1.436)	0.047	1.177(1.002-1.382)
**Fibrinogen (≥3.6/<3.6 gL^−1^)**		0.792	1.024(0.859-1.221)	0.402	1.079(0.904-1.287)
**LMR (≥3.6/<3.6)**		0.464	0.873(0.607-1.256)	0.245	0.806(0.561-1.159)
**PLT (≥300/<300 ×109L^−1^)**		0.100	1.168(0.969-1.400)	0.125	1.150(0.960-1.393)
**COP-LMR (1, 2/0)**		<0.001	1.789(1.311-2.445)	<0.001	1.919(1.404-2.618)

Abbreviations: DFS, disease-free survival; OS, overall survival; NSCLC, non-small cell lung cancer; HR, hazard ratio; CI, confidence interval; LDH, lactate dehydrogenase; ALP, alkaline phosphatase; Hb, hemoglobin; WBC, white blood cell; COP-LMR, combination of preoperative platelet count and lymphocyte to monocyte ratio. HR was calculated with reference to the last category. *P* value <0.05 is statistically significant.

**Figure 2:**
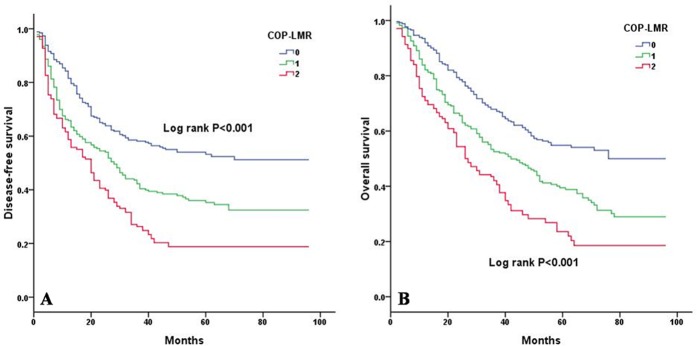
Kaplan-Meier curves for NSCLC patients according to COP-LMR levels **A.** Kaplan-Meier curve of DFS for NSCLC patients (log-rank, *P* < 0.001). **B.** Kaplan-Meier curve of OS for NSCLC patients (log-rank, *P* < 0.001).

**Figure 3:**
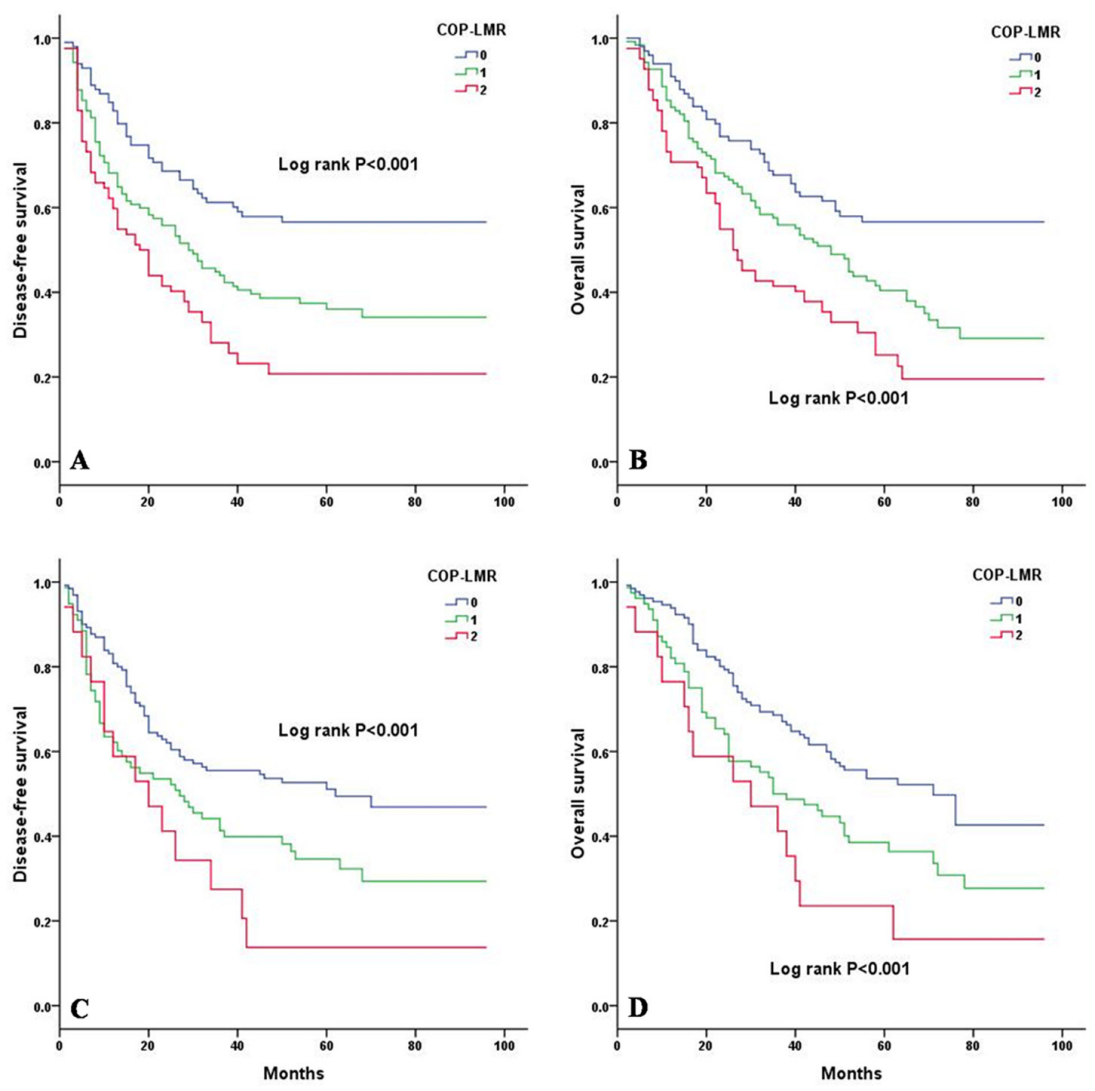
Kaplan-Meier curves for SqCC and adenocarcinoma patients according to COP-LMR levels **A.** Kaplan-Meier curve of DFS for SqCC patients (log-rank, *P* < 0.001). **B.** Kaplan-Meier curve of OS for SqCC patients (log-rank, *P* < 0.001). **C.** Kaplan-Meier curve of DFS for adenocarcinoma patients (log-rank, *P* < 0.001). **D.** Kaplan-Meier curve of OS for adenocarcinoma patients (log-rank, *P* < 0.001).

**Figure 4:**
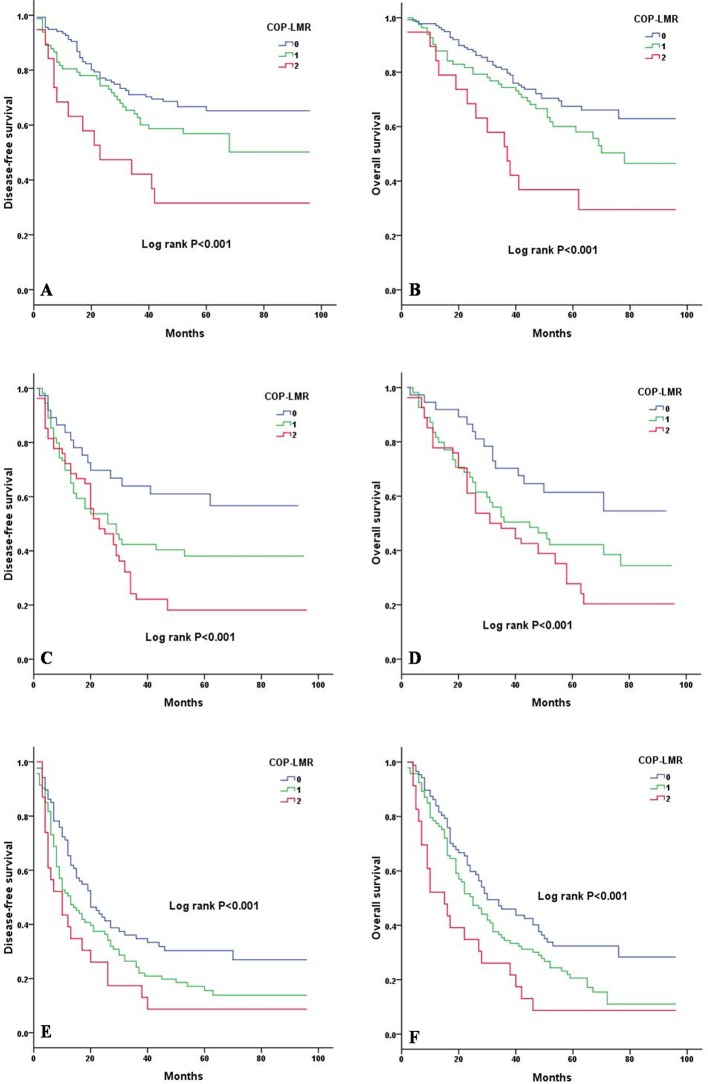
Kaplan-Meier curves of stage I, II and IIIA NSCLC patients according to COP-LMR levels **A.** Kaplan-Meier curve of DFS for stage I NSCLC patients (log-rank, *P* < 0.001). **B.** Kaplan-Meier curve of OS for stage I NSCLC patients (log-rank, *P* < 0.001). **C.** Kaplan-Meier curve of DFS for stage II NSCLC patients (log-rank, *P* < 0.001). **D.** Kaplan-Meier curve of OS for stage II NSCLC patients (log-rank, *P* < 0.001). **E**. Kaplan-Meier curve of DFS for stage IIIA NSCLC patients (log-rank, *P* < 0.001). **F**. Kaplan-Meier curve of OS for stage IIIA NSCLC patients (log-rank, *P* < 0.001).

Kaplan-Meier analysis and log-rank test showed significant differences in DFS and OS among the three COP-LMR groups (*P* < 0.001 for DFS and *P* < 0.001 for OS) (Figure 2A and 2B). Patients with COP-LMR = 2 were inclined to have worse survival than those with COP-LMR = 0 or COP-LMR = 1. Furthermore, the 5-year survival rates for patients with COP-LMR = 0, 1, and 2 were 54.8%, 39.4%, and 21.9%, respectively. Therefore, COP-LMR clearly classified NSCLC patients into 3 independent groups before surgery. Likewise, our study showed that COP-LMR clearly divided the patients into 3 independent groups in SqCC or adenocarcinoma (Figure 3A-3D). Moreover, among the patients with tumors of pathological stages I, II, and IIIA, subgroup analyses indicated that the patients with COP-LMR = 2 had a poorer survival rate than patients with COP-LMR = 0 and COP-LMR = 1 (Figure 4A-4F).

## DISCUSSION

Over the past years, a growing number of studies have investigated the clinical utility of systemic inflammation-based prognostic systems in patients with several types of cancer [5]. Such systems are useful for predicting the survival rates of cancer patients and are able to classify cancer patients before surgery.

As dynamic reservoirs of various factors, platelets secrete a number of cytokines and growth factors from granules and lysosomes, which affect migration and proliferation, epithelial to mesenchymal transition, and angiogenic activity [12]. Platelets are a source of platelet-derived growth factor, which contributes to tumor cell proliferation and angiogenesis [13]. Platelet-derived TGF augments tumor metastasis by promoting epithelial to mesenchymal transition through the activation of the Smad and NF-kB pathways [14]. Tumor cells can also produce the thrombopoietic cytokine interleukin-6, which affects cell proliferation and stimulates the differentiation of megakaryocytes to platelets in the bone marrow [15, 16]. Moreover, tumor cells can induce platelet aggregation and lead to the so-called tumor cell-induced platelet aggregation, which allows tumor cells to escape from immune surveillance [17]. According to the above results, recent studies have investigated the prognostic value of PLT in a variety of tumors, including NSCLC, and found that PLT can predict prognosis [9, 18]. With respect to the platelet count, several previous studies used cut-off values of 30×104 mm-3 to 40×104 mm-3 [19, 20]. Although 30.0×104 mm-3 is the maximum normal value for platelet count, the cut-off value for reactive thrombocytosis is not clearly defined. In our study, the ROC curve showed a cut-off value of 29.5 ×104 mm-3 for platelet count. Therefore, 30.0×104 mm-3 is an acceptable cut-off value for platelet count.

LMR is a measure of the relative difference between lymphocyte and monocyte counts and is an index of systemic inflammation. The immune response of the host to the cancer is lymphocyte dependent. Patients with relative lymphocytopenia might have a poorer lymphocyte-mediated immune response to cancer, thereby increasing the potential for cancer progression and worsening outcomes. Similarly, elevated monocytes have been correlated with poor survival in several malignancies [21–23]. Increasing evidence revealed that in a wide spectrum of tumors, a high density of tumor-associated macrophages, which are derived from monocytic precursors circulating in blood, are associated with angiogenesis, invasiveness, and poor outcomes [24]. By secreting various proteases and protease activators [25], macrophages can degrade the extracellular matrix, thus facilitating migration and invasion. This phenomenon explains why an elevated monocyte count leads to poor prognosis in patients with tumors. The prognostic value of LMR in lung cancer has already been investigated [8, 26–29]. The cut-off value of LMR in NSCLC varies from 3.29 to 4.56 [8, 27–29]. By using ROC curve, the cut-off value of LMR was determined to be 3.6 in our study, which is comparable with that in previous studies [8, 27–29].

According to recent findings, both PLT and LMR might have important associations with tumor progression. COP-LMR was derived from attempts to validate the stratification of patients by using reactive thrombocytosis and cellular inflammation-based prognostic markers, such as LMR. A higher proportion of patients with COP-LMR = 1 or 2 tend to have larger tumors than patients with COP-LMR = 0 because tumor size reflects tumor progression and malignancy. ALP is a tumor-associated antigen. High ALP activities or changes in the nucleus during the cell cycles reflect tumor cell proliferation and progression [30]. Hypoalbuminemia and low Hb are related to poor nutritional status, which reflect cachexia resulting from cancer progression. As a major acute-phase protein, increased fibrinogen is probably induced by an inflammatory response to cancer progression and a prothrombotic state in cancer patients [31]. Cancer cells can release procoagulant molecules and proinflammatory cytokines to activate the coagulation system [32]. D-dimer is a marker of the activation of coagulation and fibrinolysis. Thus, significant differences were observed in ALP, fibrinogen, D-dimer, albumin and Hb levels among the three COP-LMR groups.

In our study, univariate analysis was used to identify 18 variables related to systemic inflammatory response or tumor. Ultimately, 13 variables were entered into a multivariate analysis. These variables showed significant relationships between COP-LMR and both DFS and OS, along with pathological stage, Hb and D-dimer. Based on the Kaplan-Meier analysis and log-rank test, our results indicated that preoperative COP-LMR is capable of stratifying patients into 3 independent groups. Furthermore, our study also revealed that COP-LMR acts as an independent prognostic factor in SqCC or adenocarcinoma patients. We also separately assessed the prognostic value of COP-LMR in patients with stage I, II and IIIA tumors, and our results showed that COP-LMR level was significantly associated with DFS and OS in stages I, II and IIIA tumors. The results of survival analysis in histological subtypes or pathological subgroups were consistent with the outcomes of the survival analysis on the whole study population, thus indicating that our findings were reliable.

Although our study is the first to investigate the prognostic value of COP-LMR in NSCLC patients who underwent surgery, we must recognize that the present study has several potential limitations. First, the major limitation of this study includes the use of retrospective analysis and single-center management; we cannot fully exclude selection bias and control for all possible risk factors for cancer patients. Second, although we tried to reduce the effect of other factors, lymphocyte and monocyte counts can be influenced by several factors. In the future, prospective clinical studies are needed to confirm our results and promote the clinical application of COP-LMR. Preclinical studies are also needed to provide the basis for clinical application.

COP-LMR is easy to routinely assess because of its low cost and convenience. Moreover, repeat measurements of COP-LMR can be easily performed before and after surgery. Sequential follow-up using COP-LMR would be a useful adjunct to imaging examinations, such as computed tomography and magnetic resonance imaging.

In summary, our study showed that preoperative COP-LMR is capable of dividing NSCLC patients into 3 independent groups before surgery and shows potential as a novel prognostic factor for postoperative survival in these patients.

## MATERIALS AND METHODS

### Patients

Patients were selected from a retrospective cohort of 1408 patients newly diagnosed with NSCLC between January 2007 and December 2011 at the First Affiliated Hospital of Jinzhou Medical University. The inclusion criteria consist of the following: (1) histologically confirmed NSCLC and (2) complete pulmonary resection and systematic node dissection of the hilar and mediastinal lymph nodes. The exclusion criteria included the following: (1) patients who previously received preoperative chemotherapy or radiotherapy, (2) patients without data of PLT or LMR, (3) patients with advanced disease (e.g., malignant pleural effusion/involvement or distant metastasis), and (4) patients with clinical evidence of infection or other bone marrow, hematological, or autoimmune disease. Based on the inclusion and exclusion criteria, a total of 1120 patients were enrolled in the present study. The seventh edition of the TNM classification system was used in this retrospective study [33]. This study was approved by the ethics committee of the First Affiliated Hospital of Jinzhou Medical University. All patients signed informed consent.

### Definition of COP-LMR and other variables

Venous blood samples were obtained at one week prior to surgery and collected in ethylenediaminetetraacetic acid (EDTA)-containing tubes. LMR was calculated as follows: LMR = peripheral lymphocyte count/peripheral monocyte count. The cut-off values of the preoperative PLT and LMR were decided by ROC curve analysis. COP-LMR was calculated on the basis of the obtained data. Patients with both an increased PLT ( > 30×104 mm-3) and a low LMR ( < 3.6) were assigned a score of 2, and patients with one or none of the parameters were assigned a score of 1 or 0, respectively.

For the eligible patients, clinicopathological and clinicolaboratory information including sex and age at diagnosis, smoking status, tumor location, lesion type, resection type, histological subtype, lymph node metastasis, maximum tumor diameter, pathological stage, WBC count, PLT, LMR, Hb, albumin, LDH, ALP, D-dimer, fibrinogen, and COP-LMR was collected for subsequent analysis. The cut-off values of clinicolaboratory variables were determined by ROC curve analysis to classify the patients into two groups. The values of maximum joint sensitivity and specificity on the ROC plot were defined as the recommended cut-off value. The cut-off values for WBC count, Hb, albumin, LDH, ALP, D-dimer, and fibrinogen were 7.8×103 mm-3, 130.5 gL-1, 44.9 gL-1, 174 UL-1, 71.0 UL-1, 0.1 mgL-1, and 3.6 gL-1, respectively.

### Statistical analysis

SPSS 13.0 (SPSS Inc., Chicago, USA) was used for all statistical analyses. DFS was defined as the time between the date of surgery and the date of first recurrence or last follow-up. OS was defined as the interval between the date of surgery and the date of death or last follow-up. The data are presented as the mean ± s.d. For normally distributed results, one-way analysis of variance (ANOVA) was used to compare the clinicolaboratory variables between the 3 COP-LMR groups. The differences between the 3 COP-LMR groups and clinicopathological variables were analyzed using chi-square test. Survival curves were generated by the Kaplan-Meier method, and differences between survival curves were estimated by log-rank test. HR and 95% CI were calculated by univariate and multivariate analyses using the Cox proportional hazards model. Comparisons between the values for AUC were accomplished with the nonparametric approach established by DeLong et al. [11]. *P* value < 0.05 was considered significant.

## SUPPLEMENTARY MATERIALS FIGURES AND TABLES










