# Supplementary material for: Combination of platelet count and lymphocyte to monocyte ratio is a prognostic factor in patients undergoing surgery for non-small cell lung cancer
Source: Oncotarget. 2017 Jun 1;8(42):73198–207. doi: 10.18632/oncotarget.18336 (PMC5641206; doi:10.18632/oncotarget.18336)
Supplement: Supplementary file 1 [file oncotarget-08-73198-s001.pdf]

Combination of platelet count and lymphocyte to monocyte ratio is a prognostic factor in patients undergoing surgery for non-small cell lung cancer

Supplementary Material

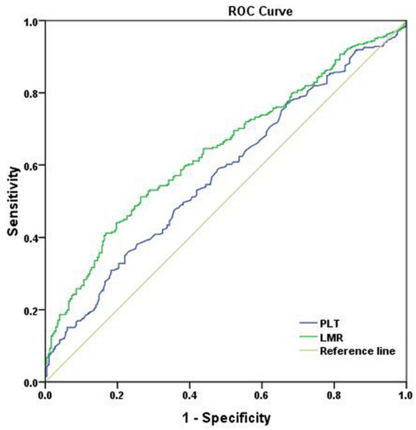

Supplementary Figure 1: ROC curves for survival prediction. ROC curves were plotted to verify the accuracy of PLT and LMR for survival. The AUC was 0.576 for PLT and 0.641 for LMR.

For Supplementary Tables see in Supplementary Files.
