# Supplementary material for: Combination of platelet count and lymphocyte to monocyte ratio is a prognostic factor in patients undergoing surgery for non-small cell lung cancer
Source: Oncotarget. 2017 Jun 1;8(42):73198–207. doi: 10.18632/oncotarget.18336 (PMC5641206; doi:10.18632/oncotarget.18336)
Supplement: Supplementary file 2 [file oncotarget-08-73198-s002.docx]

**Supplementary Table S1. Univariate** **analysis for DFS and OS for squamous cell carcinoma patients.**

| Variables |  | *P*  value | DFS  HR (95 % CI) | *P* value | OS  HR (95 % CI) |
| --- | --- | --- | --- | --- | --- |
| Age (≤60/>60) | | 0.110 | 0.831(0.663-1.043) | 0.096 | 0.825(0.658-1.034) |
| Sex (female/male) |  | 0.442 | 0.890(0.660-1.199) | 0.355 | 0.869(0.644-1.171) |
| Smoking status (yes/no) | | 0.513 | 1.110(0.811-1.520) | 0.600 | 1.088(0.794-1.489) |
| Tumor location (left/right) | | 0.252 | 1.145(0.908-1.443) | 0.238 | 1.150(0.912-1.449) |
| Lesion type (central/peripheral) | | 0.090 | 1.216(0.970-1.525) | 0.105 | 1.225(0.975-1.531) |
| Resection type (pneumonectomy/lobectomy) | | 0.349 | 1.139(0.867-1.497) | 0.489 | 1.101(0.838-1.446) |
| Pathological stage (IIIA/I, II) | | <0.001 | 2.418(1.923-3.042) | <0.001 | 2.394(1.903-3.012) |
| LDH (≥174.0/<174.0 UL^-1^) | | 0.167 | 1.172(0.936-1.469) | 0.253 | 1.141(0.910-1.429) |
| ALP (≥71.0/<71.0 UL^-1^) | | 0.048 | 1.257(1.002-1.576) | 0.043 | 1.264(1.008-1.585) |
| Hb ≥130.5/<130.5 gL^-1^) | | <0.001 | 0.541(0.423-0.691) | <0.001 | 0.540(0.422-0.690) |
| Albumin (≥44.9/<44.9 gL^-1^) | | 0.012 | 0.704(0.536-0.925) | 0.016 | 0.715(0.544-0.940) |
| WBC count(≥7.8/<7.8× 10^3^ mm^-3^) | | 0.013 | 1.337(1.062-1.683) | 0.045 | 1.265(1.005-1.592) |
| PLT (≥300/<300 ×10^9^L^-1^) | | 0.001 | 1.508(1.179-1.931) | <0.001 | 1.497(1.171-1.916) |
| D-dimer (≥0.1/<0.1 mgL^-1^) | | <0.001 | 1.672(1.327-2.107) | <0.001 | 1.584(1.257-1.998) |
| Fibrinogen (≥3.6/<3.6 gL^-1^) | | 0.003 | 1.441(1.137-1.827) | 0.002 | 1.443(1.138-1.830) |
| LMR (≥3.6/<3.6) | | <0.001 | 0.520(0.411-0.658) | <0.001 | 0.546(0.432-0.691) |
| COP-LMR (1, 2/0) | | <0.001 | 1.960(1.524-2.522) | <0.001 | 1.856(1.443-2.388) |

Abbreviations: DFS, disease-free survival; OS, overall survival; HR, hazard ratio; CI, confidence interval; LDH, lactate dehydrogenase; ALP, alkaline phosphatase; Hb, hemoglobin; WBC, white blood cell; PLT, platelet count; LMR, lymphocyte to monocyte ratio; COP-LMR, combination of preoperative platelet count and lymphocyte to monocyte ratio. HR was calculated with reference to the last category. *P* value < 0.05 is statistically significant.
