# Supplementary material for: Combination of platelet count and lymphocyte to monocyte ratio is a prognostic factor in patients undergoing surgery for non-small cell lung cancer
Source: Oncotarget. 2017 Jun 1;8(42):73198–207. doi: 10.18632/oncotarget.18336 (PMC5641206; doi:10.18632/oncotarget.18336)
Supplement: Supplementary file 3 [file oncotarget-08-73198-s003.docx]

**Supplementary Table S2. Multivariate analysis for DFS and OS for squamous cell carcinoma patients.**

| Variables |  | *P* value | DFS  HR (95 % CI) | *P* value | OS  HR (95 % CI) |
| --- | --- | --- | --- | --- | --- |
| Pathological stage (IIIA/I, II) | | <0.001 | 2.556(2.020-3.234) | <0.001 | 2.542(2.005-3.224) |
| ALP (≥71.0/<71.0 UL^-1^) | | 0.661 | 1.054(0.834-1.330) | 0.558 | 1.072(0.849-1.355) |
| Hb (≥130.5/<130.5 gL^-1^) | | 0.001 | 0.644(0.494-0.839) | 0.001 | 0.623(0.476-0.815) |
| Albumin (≥44.9/<44.9 gL^-1^) | | 0.168 | 0.813(0.606-1.091) | 0.271 | 0.847(0.631-1.138) |
| WBC count (≥7.8/<7.8× 10^3^ mm^-3^) | | 0.096 | 1.231(0.964-1.572) | 0.390 | 1.114(0.870-1.427) |
| D-dimer (≥0.1/<0.1 mgL^-1^) | | 0.007 | 1.396(1.096-1.778) | 0.016 | 1.343(1.056-1.708) |
| Fibrinogen (≥3.6/<3.6 gL^-1^) | | 0.706 | 1.052(0.810-1.366) | 0.559 | 1.082(0.830-1.411) |
| LMR (≥3.6/<3.6) | | 0.412 | 0.797(0.464-1.370) | 0.266 | 0.734(0.426-1.266) |
| PLT (≥300/<300 ×10^9^L^-1^) | | 0.098 | 1.307(0.952-1.795) | 0.079 | 1.406(0.991-1.869) |
| COP-LMR (1, 2/0) | | 0.001 | 2.070(1.300-3.289) | 0.001 | 2.058(1.290-3.279) |

Abbreviations: DFS, disease-free survival; OS, overall survival; HR, hazard ratio; CI, confidence interval; ALP, alkaline phosphatase; Hb, hemoglobin; WBC, white blood cell; COP-LMR, combination of preoperative platelet count and lymphocyte to monocyte ratio. HR was calculated with reference to the last category. *P* value <0.05 is statistically significant.
