# Supplementary material for: Combination of platelet count and lymphocyte to monocyte ratio is a prognostic factor in patients undergoing surgery for non-small cell lung cancer
Source: Oncotarget. 2017 Jun 1;8(42):73198–207. doi: 10.18632/oncotarget.18336 (PMC5641206; doi:10.18632/oncotarget.18336)
Supplement: Supplementary file 4 [file oncotarget-08-73198-s004.docx]

**Supplementary Table S3. Univariate** **analysis for DFS and OS for adenocarcinoma patients.**

| Variables |  | *P*  value | DFS  HR (95 % CI) | *P* value | OS  HR (95 % CI) |
| --- | --- | --- | --- | --- | --- |
| Age (≤60/>60) | | 0.849 | 0.976(0.760-1.253) | 0.902 | 1.016(0.791-1.304) |
| Sex (female/male) |  | 0.326 | 0.884(0.691-1.131) | 0.127 | 0.825(0.645 -1.056) |
| Smoking status (yes/no) | | 0.012 | 1.368(1.070 -1.748) | 0.001 | 1.499(1.172- 1.916) |
| Tumor location (left/right) | | 0.852 | 1.025(0.794-1.321) | 0.864 | 1.022(0.793-1.318) |
| Lesion type (central/peripheral) | | 0.312 | 1.186(0.852-1.652) | 0.369 | 1.164(0.836-1.623) |
| Resection type (pneumonectomy/lobectomy) | | 0.120 | 1.534(0.894 -2.629) | 0.065 | 1.662(0.968-2.852) |
| Pathological stage (IIIA/I, II) | | <0.001 | 2.271(1.773-2.908) | <0.001 | 2.245(1.754-2.873) |
| LDH (≥174.0/<174.0 UL^-1^) | | 0.264 | 1.150(0.900-1.469) | 0.237 | 1.160(0.907-1.482) |
| ALP (≥71.0/<71.0 UL^-1^) | | 0.196 | 1.176(0.920-1.503) | 0.077 | 1.248(0.976 -1.595) |
| Hb (≥130.5/<130.5 gL^-1^) | | 0.009 | 0.707(0.545-0.919) | 0.018 | 0.729(0.561-0.964) |
| Albumin (≥44.9/<44.9 gL^-1^) | | <0.001 | 0.630(0.486-0.816) | <0.001 | 0.646(0.498-0.836) |
| WBC count(≥7.8/<7.8× 10^3^ mm^-3^) | | 0.002 | 1.556(1.172-2.066) | 0.001 | 1.735(1.306 -2.304) |
| PLT (≥300/<300 ×10^9^L^-1^) | | 0.037 | 1.328(1.049-1.779) | 0.035 | 1.332(1.053 -1.795) |
| D-dimer (≥0.1/<0.1 mgL^-1^) | | 0.017 | 1.351(1.055-1.729) | 0.034 | 1.308(1.020-1.677) |
| Fibrinogen (≥3.6/<3.6 gL^-1^) | | <0.001 | 1.605(1.245-2.070) | <0.001 | 1.674(1.297-2.159) |
| LMR (≥3.6/<3.6) | | <0.001 | 0.532(0.414 -0.685) | <0.001 | 0.516(0.401-0.665) |
| COP-LMR (1, 2/0) | | <0.001 | 1.824(1.449-2.304) | <0.001 | 1.845(1.465-2.330) |

Abbreviations: DFS, disease-free survival; OS, overall survival; HR, hazard ratio; CI, confidence interval; LDH, lactate dehydrogenase; ALP, alkaline phosphatase; Hb, hemoglobin; WBC, white blood cell; PLT, platelet count; LMR, lymphocyte to monocyte ratio; COP-LMR, combination of preoperative platelet count and lymphocyte to monocyte ratio. HR was calculated with reference to the last category. *P* value <0.05 is statistically significant.
