# Supplementary material for: Combination of platelet count and lymphocyte to monocyte ratio is a prognostic factor in patients undergoing surgery for non-small cell lung cancer
Source: Oncotarget. 2017 Jun 1;8(42):73198–207. doi: 10.18632/oncotarget.18336 (PMC5641206; doi:10.18632/oncotarget.18336)
Supplement: Supplementary file 5 [file oncotarget-08-73198-s005.docx]

**Supplementary Table S4. Multivariate analysis for DFS and OS for adenocarcinoma patients.**

| Variables |  | *P* value | DFS  HR (95 % CI) | *P* value | OS  HR (95 % CI) |
| --- | --- | --- | --- | --- | --- |
| Smoking status (yes/no) | | 0.013 | 1.403(1.075 -1.832) | 0.001 | 1.546(1.182-2.020) |
| Pathological stage (IIIA/I, II) | | <0.001 | 1.994(1.537-2.587) | <0.001 | 2.035(1.574-2.631) |
| Hb (≥130.5/<130.5 gL^-1^) | | 0.008 | 0.683(0.514- 0.907) | 0.002 | 0.640(0.482-0.851) |
| Albumin (≥44.9/<44.9 gL^-1^) | | 0.097 | 0.792(0.601-1.043) | 0.116 | 0.802(0.610-1.056) |
| WBC count (≥7.8/<7.8× 10^3^ mm^-3^) | | 0.185 | 1.228(0.907-1.663) | 0.150 | 1.255(0.912-1.670) |
| D-dimer (≥0.1/<0.1 mgL^-1^) | | 0.225 | 1.173(0.906-1.518) | 0.280 | 1.154(0.890-1.496) |
| Fibrinogen (≥3.6/<3.6 gL^-1^) | | 0.117 | 1.245(0.946- 1.639) | 0.090 | 1.271(0.972-1.667) |
| LMR (≥3.6/<3.6) | | 0.439 | 0.789(0.432-1.438) | 0.236 | 0.696(0.382-1.267) |
| PLT (≥300/<300 ×10^9^L^-1^) | | 0.176 | 1.361(0.870-2.128) | 0.090 | 1.471(0.961-2.293) |
| COP-LMR (1, 2/0) | | 0.025 | 1.742(1.033-2.933) | 0.007 | 2.058(1.222-3.460) |

Abbreviations: DFS, disease-free survival; OS, overall survival; HR, hazard ratio; CI, confidence interval; Hb, hemoglobin; WBC, white blood cell; COP-LMR, combination of preoperative platelet count and lymphocyte to monocyte ratio. HR was calculated with reference to the last category. *P* value <0.05 is statistically significant.
